# Supplementary material for: Molecular basis promoting centriole triplet microtubule assembly
Source: Nat Commun. 2024 Mar 22;15:2216. doi: 10.1038/s41467-024-46454-x (PMC10960023; doi:10.1038/s41467-024-46454-x)
Supplement: Supplementary file 1 — Supplementary Information [file 41467_2024_46454_MOESM1_ESM.pdf]

Supplementary Information

Molecular basis promoting centriole triplet microtubule assembly

Yutaka Takeda, Takumi Chinen, Shunnosuke Honda, Sho Takatori, Shotaro Okuda, Shohei Yamamoto, Masamitsu Fukuyama, Koh Takeuchi, Taisuke Tomita, Shoji Hata & Daiju Kitagawa

*Corresponding authors, e-mail: takumi.chinen@mol.f.u-tokyo.ac.jp (Takumi Chinen), s.hata@mol.f.u-tokyo.ac.jp (Shoji Hata), dkitagawa@mol.f.u-tokyo.ac.jp (Daiju Kitagawa).

Supplementary Fig. 1

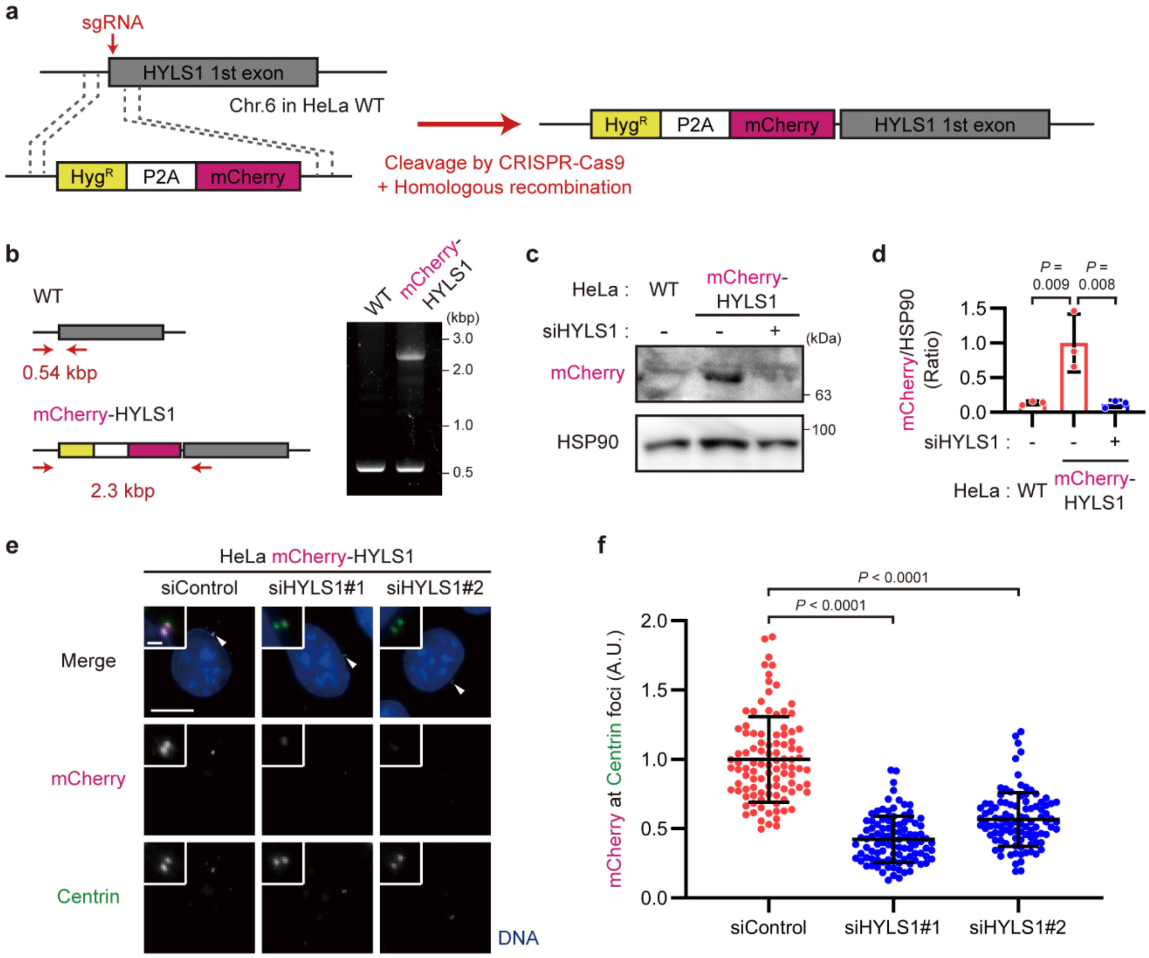

Supplementary Fig. 1 Generation of HeLa mCherry-HYLS1 knock-in cells.

a, Schematic of CRISPR-mediated knock-in method to generate HeLa mCherry-HYLS1 knock-in cells. Chr.: chromosome, HygR: hygromycin resistance gene. **b**, Genomic PCR and agarose gel electrophoresis of HeLa wildtype (WT) and HeLa mCherry-HYLS1 cells using the indicated primers. **c**, Immunoblotting images of lysates from HeLa WT and HeLa mCherry-HYLS1 cells treated with siControl or siHYLS1. **d**, Quantification of the intensity of mCherry normalized to that of HSP90 in **c**. n =3 independent experiments. **e**, IF images of HeLa mCherry-HYLS1 cells treated with siControl or siHYLS1. Scale bars: 10 μm and 1 μm. Arrowheads: magnified areas. **f**, Quantification of the intensity of mCherry at Centrin foci (centrioles) in **e**. A.U.: arbitrary unit. n = 100 cells. Data are represented as mean ± s.d.. P values were calculated by one-way ANOVA with Dunnett's multiple comparisons test (**d**) or Dunn's multiple comparisons test (**f**). Source data are provided as a Source Data file.

Supplementary Fig. 2

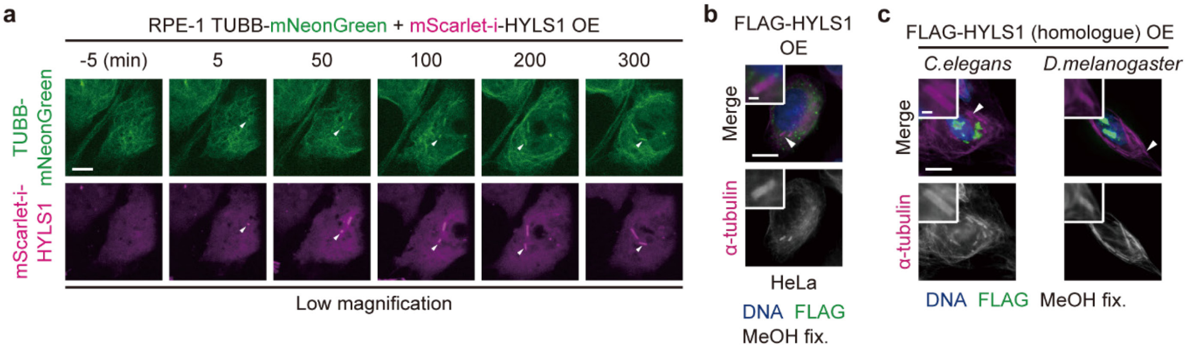

Supplementary Fig. 2 Overexpression of the ciliopathy protein HYLS1 leads to assembly of tubulin-based superstructures.

a, Supplementary time-lapse images related to Fig. 1h. Scale bar: 10 µm. Arrowheads: magnified areas in Fig. 1h. **b**, IF images of HeLa cells transfected with pCMV-FLAG-HYLS1. Scale bars: 10 µm and 1 µm. Arrowhead: magnified area. **c**, IF images of RPE-1 cells transfected with pCMV-FLAG-HYLS1 homologues of *C. elegans* or *D. melanogaster*. Scale bars: 10 µm and 1 µm. Arrowheads: magnified areas.

Supplementary Fig. 3

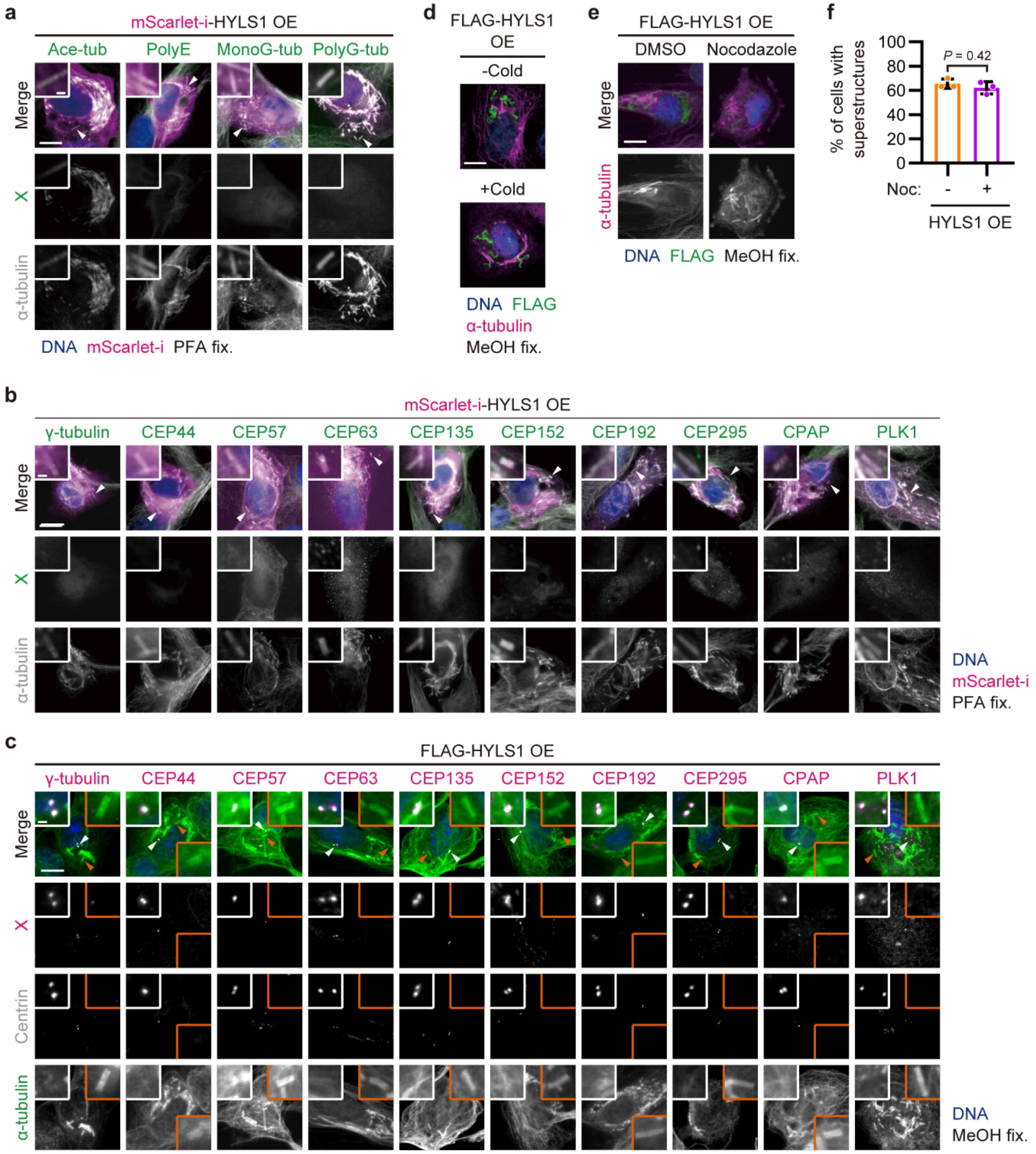

Supplementary Fig. 3 Overexpression of HYLS1 leads to assembly of tubulin-based superstructures with high stability similar to that of centrioles.

a, **b**, IF images of RPE-1 cells transfected with pCMV-mScarlet-i-HYLS1 and fixed with PFA. Scale bars: 10 µm and 1 µm. Arrowheads: magnified areas. Ace-tub: acetylated tubulin, PolyE: polyglutamylation modification, MonoG-tub: monoglycylated tubulin, PolyG-tub: polyglycylated

tubule. **c**, IF images of RPE-1 cells transfected with pCMV-mScarlet-i-HYLS1 and fixed with MeOH. Scale bars: 10 μm and 1 μm. Arrowheads: magnified areas including centrioles (white) or superstructures (orange). **d**, Supplementary IF images related to Fig. 1k. Scale bar: 10 μm. **e**, IF images of RPE-1 cells transfected with pCMV-FLAG-HYLS1 and then treated with DMSO or nocodazole for 2 hours before fixation. Scale bar: 10 μm. **f**, Quantification of frequency of interphase cells with the tubulin-based superstructures in **e**. n = 3 independent experiments, 30 cells each. Data are represented as mean ± s.d.. P value was calculated by two-tailed unpaired Student's t-test (**f**). Source data are provided as a Source Data file.

Supplementary Fig. 4

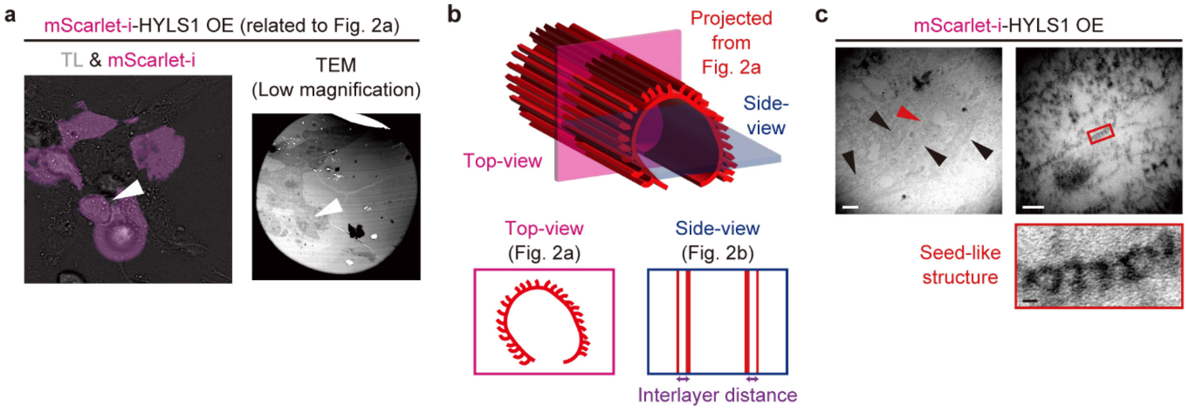

Supplementary Fig. 4 Overexpression of HYLS1 leads to assembly of tubulin-based superstructures with incomplete microtubule chains.

a, Supplementary CLEM images related to Fig. 2a. Fluorescence image of pre-fixed cells and TEM image at low magnification of them. Arrowheads: observed cell in Fig. 2a. TL: transmitted light. **b**, Schematic showing assumed geometry of the superstructure and its TEM images. **c**, TEM images of seed-like structures in RPE-1 cells transfected with pCMV-mScarlet-i-HYLS1. Scale bars: 500 nm, 100 nm, and 10 nm. Red arrowhead: observed seed-like structure, black arrowheads: superstructures.

Supplementary Fig. 5

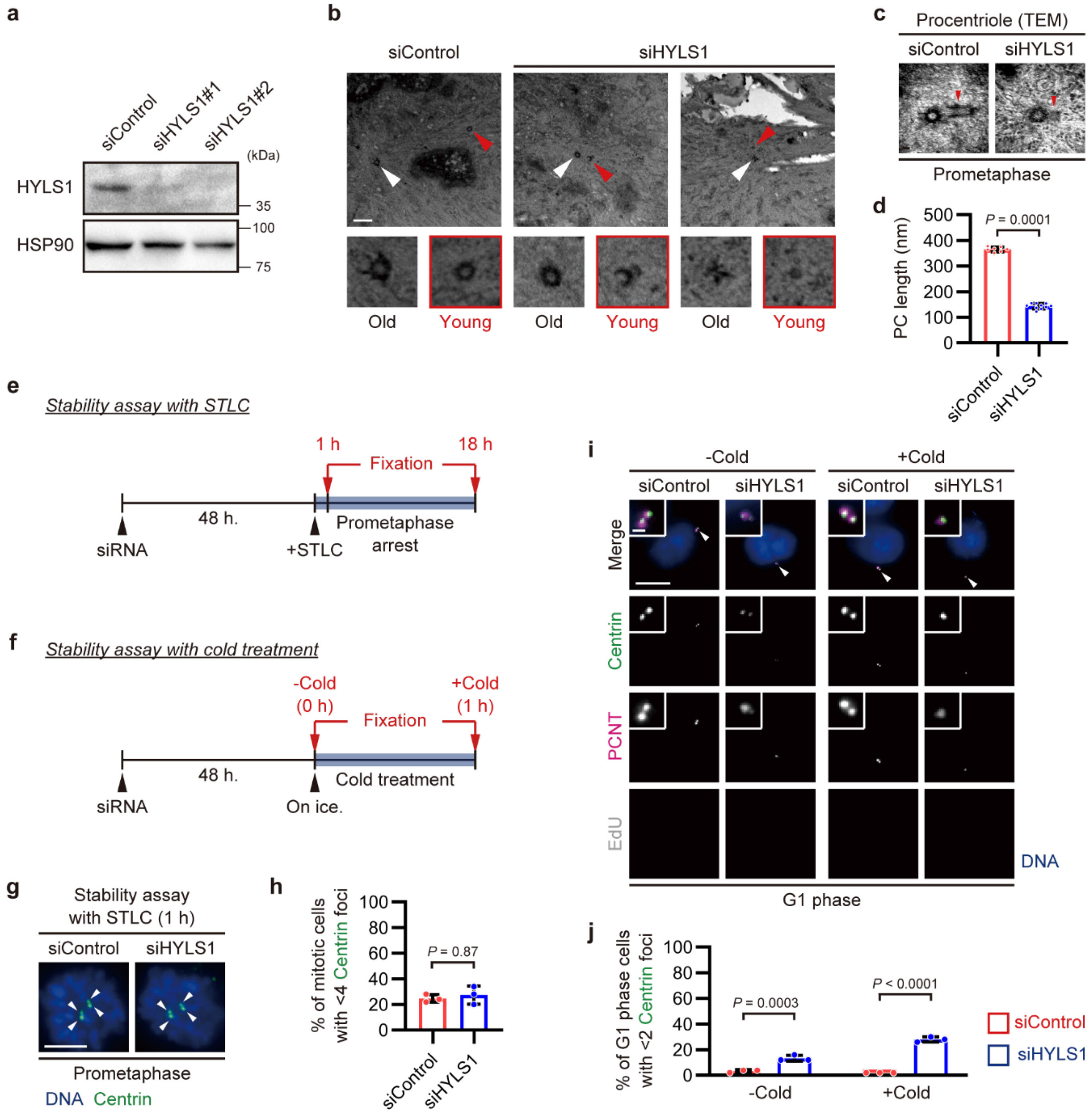

Supplementary Fig. 5 HYLS1 provides the structural integrity of centriole triplet microtubules.

a, Immunoblotting images of lysates from RPE-1 cells treated with siControl or siHYLS1. **b**, Supplementary TEM images at low magnification related to Fig. 2d. The younger centrioles were distinguished by the absence of appendages. Scale bar: 1 μm. White arrowheads: older centrioles, red arrowheads: younger centrioles. **c**, TEM images of procentrioles in prometaphase RPE-1 cells transfected with siControl or siHYLS1. To induce prometaphase arrest, the cells were treated with

STLC 3 hours before fixation. Scale bar: 100 nm. Arrowheads: procentrioles. **d**, Quantification of the length of procentrioles in **c**. *n* = 7 (siControl) or 10 (siHYLS1) cells. **e**, Time-course of centriole stability assay with STLC. **f**, Time-course of centriole stability assay with cold treatment. **g**, Supplementary IF images related to Fig. 2e. Scale bar: 5 µm. Arrowheads: Centrin foci (centrioles). **h**, Supplementary quantification of frequency of mitotic cells with <4 Centrin foci related to Fig. 2f. Groups treated with STLC for 1 hour are shown. *n* = 3 independent experiments, 50 cells each. **i**, IF images of RPE-1 cells subjected to centriole stability assay with cold treatment. Before incubation on ice, the cells were transfected with siControl or siHYLS1. Scale bars: 10 µm and 1 µm. Arrowheads: magnified areas. **j**, Quantification of frequency of G1 phase cells with <2 Centrin foci in **i**. *n* = 3 independent experiments, 50 cells each. Data are represented as mean ± s.d.. *P* values were calculated by Mann–Whitney *U* test (**d**) or one-way ANOVA with Tukey's multiple comparisons test (**h**, **j**). Source data are provided as a Source Data file.

Supplementary Fig. 6

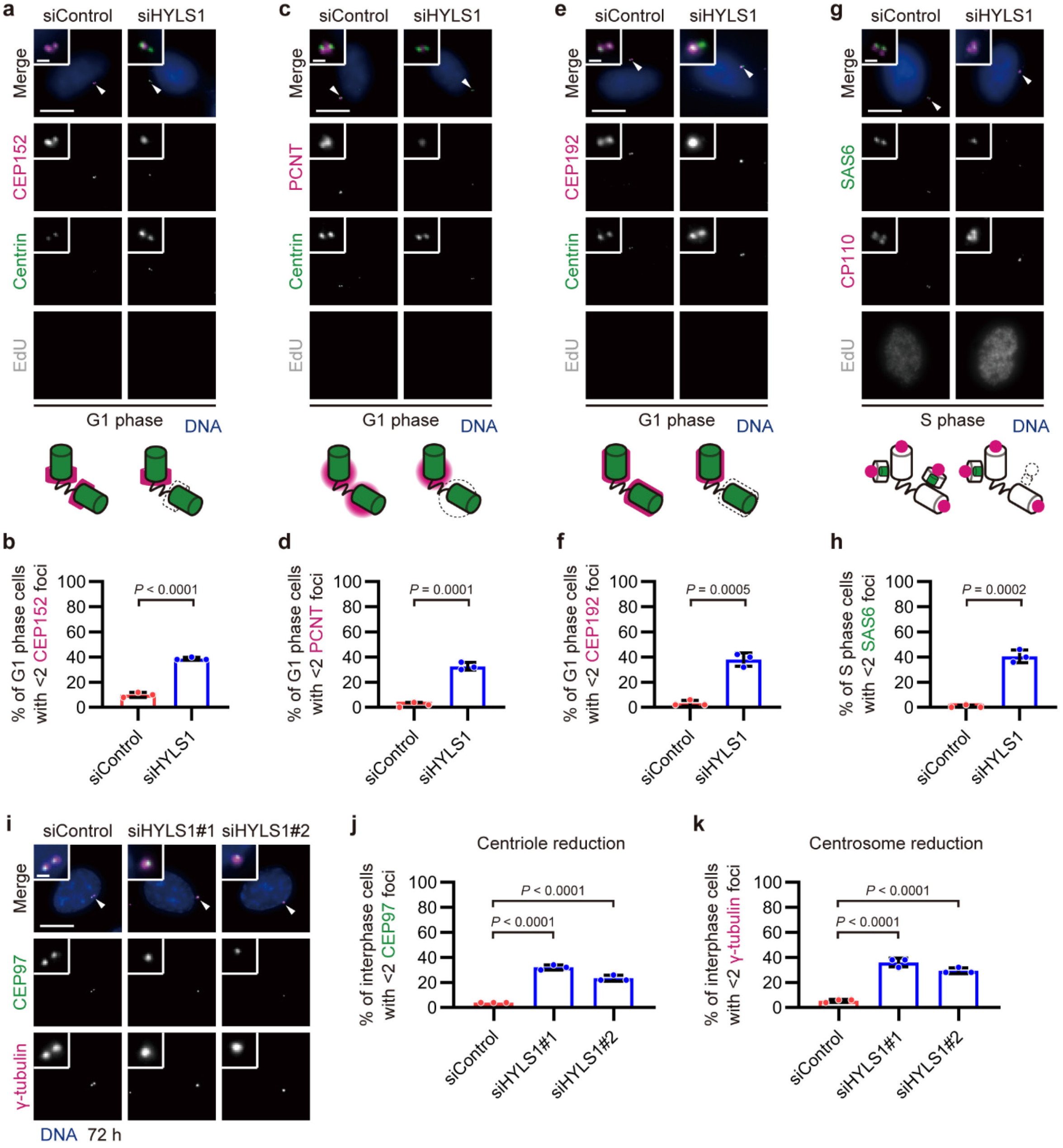

Supplementary Fig. 6 HYLS1 ensures the ability of centrioles to organize centrosomes. **a**, IF images of G1 phase RPE-1 cells transfected with siControl or siHYLS1. Scale bars: 10 μm and 1 μm. Arrowheads: magnified areas. **b**, Quantification of frequency of G1 phase cells with <2 CEP152 foci in **a**. $n = 3$ independent experiments, 50 cells each. **c**, IF images of G1 phase RPE-1 cells transfected with siControl or siHYLS1. Scale bars: 10 μm and 1 μm. Arrowheads: magnified areas. **d**, Quantification of frequency of G1 phase cells with <2 PCNT foci in **c**. $n = 3$ independent

experiments, 50 cells each. **e**, IF images of G1 phase RPE-1 cells transfected with siControl or siHYLS1. Scale bars: 10 µm and 1 µm. Arrowheads: magnified areas. **f**, Quantification of frequency of G1 phase cells with <2 CEP192 foci in **e**. $n = 3$ independent experiments, 50 cells each. **g**, IF images of S phase RPE-1 cells transfected with siControl or siHYLS1. Scale bars: 10 µm and 1 µm. Arrowheads: magnified areas. **h**, Quantification of frequency of S phase cells with <2 SAS6 foci in **g**. $n = 3$ independent experiments, 50 cells each. **i**, IF images of RPE-1 cells transfected with siControl or siHYLS1 for 72 hours. Scale bars: 10 µm and 1 µm. Arrowheads: magnified areas. **j**, Quantification of frequency of interphase cells with <2 CEP97 foci in **i**. $n = 3$ independent experiments, 50 cells each. **k**, Quantification of frequency of interphase cells with <2 γ-tubulin foci in **i**. $n = 3$ independent experiments, 50 cells each. Data are represented as mean ± s.d.. *P* values were calculated by two-tailed unpaired Student's *t*-test (**b**, **d**, **f**, **h**) or one-way ANOVA with Dunnett's multiple comparisons test (**j**, **k**). Source data are provided as a Source Data file.

Supplementary Fig. 7

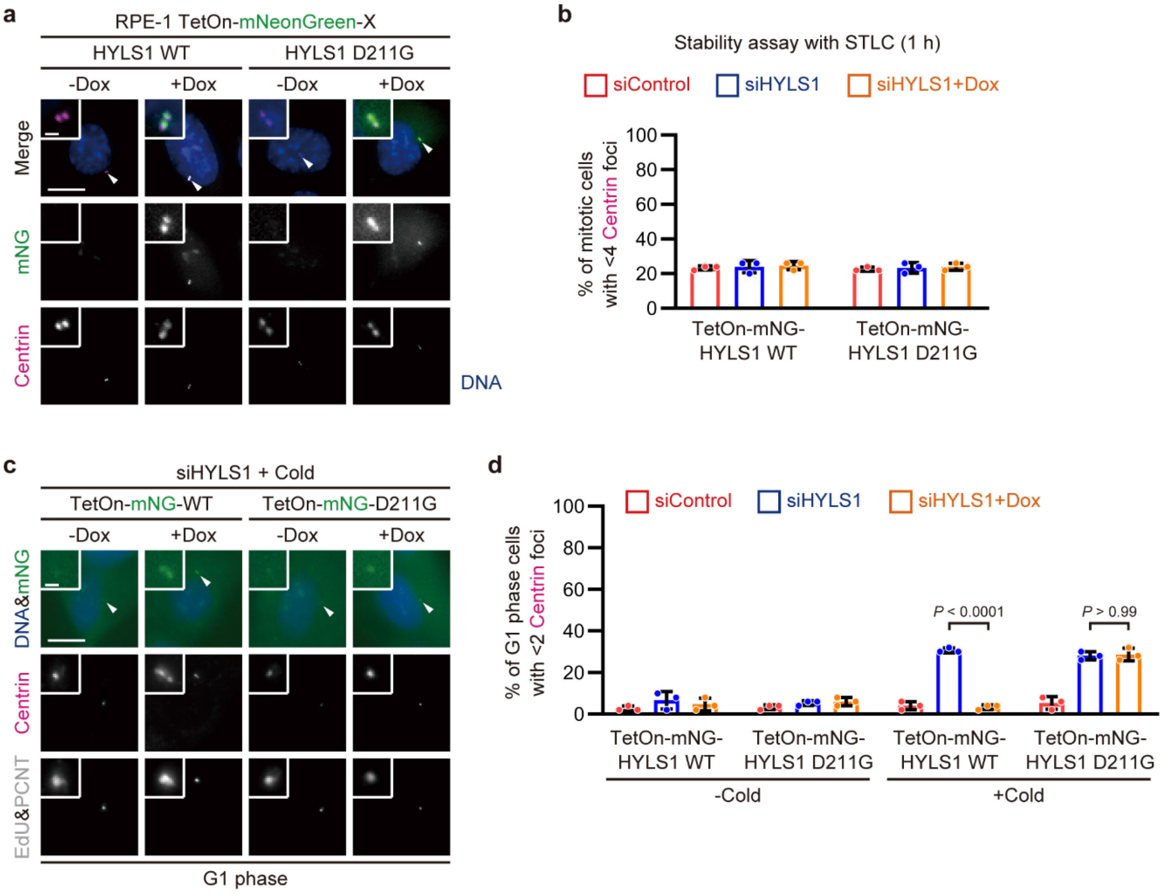

Supplementary Fig. 7 The ciliopathy-related D211G mutant of HYLS1 fails to stabilize centrioles.

a, IF images of RPE-1 TetOn-mNeonGreen-X cells (X = HYLS1 WT or D211G) in presence and absence of doxycycline. Scale bars: 10 µm and 1 µm. Arrowheads: magnified areas. **b**, Supplementary quantification of frequency of mitotic cells with <4 Centrin foci related to Fig. 3h. Groups treated with STLC for 1 hour are shown. $n = 3$ independent experiments, 50 cells each. **c**, Representative IF images of RPE-1 TetOn-mNeonGreen-X cells (X = HYLS1 WT or D211G) subjected to centriole stability assay with cold treatment. Before incubation on ice, the cells were transfected with siControl or siHYLS1 and were treated with doxycycline. Scale bars: 10 µm and 1 µm. Arrowheads: magnified areas. **d**, Quantification of frequency of G1 phase cells with <2 Centrin foci in **c**. $n = 3$ independent experiments, 50 cells each. Data are represented as mean ± s.d.. P values were calculated by one-way ANOVA with Tukey's multiple comparisons test (**d**). Source data are provided as a Source Data file.

Supplementary Fig. 8

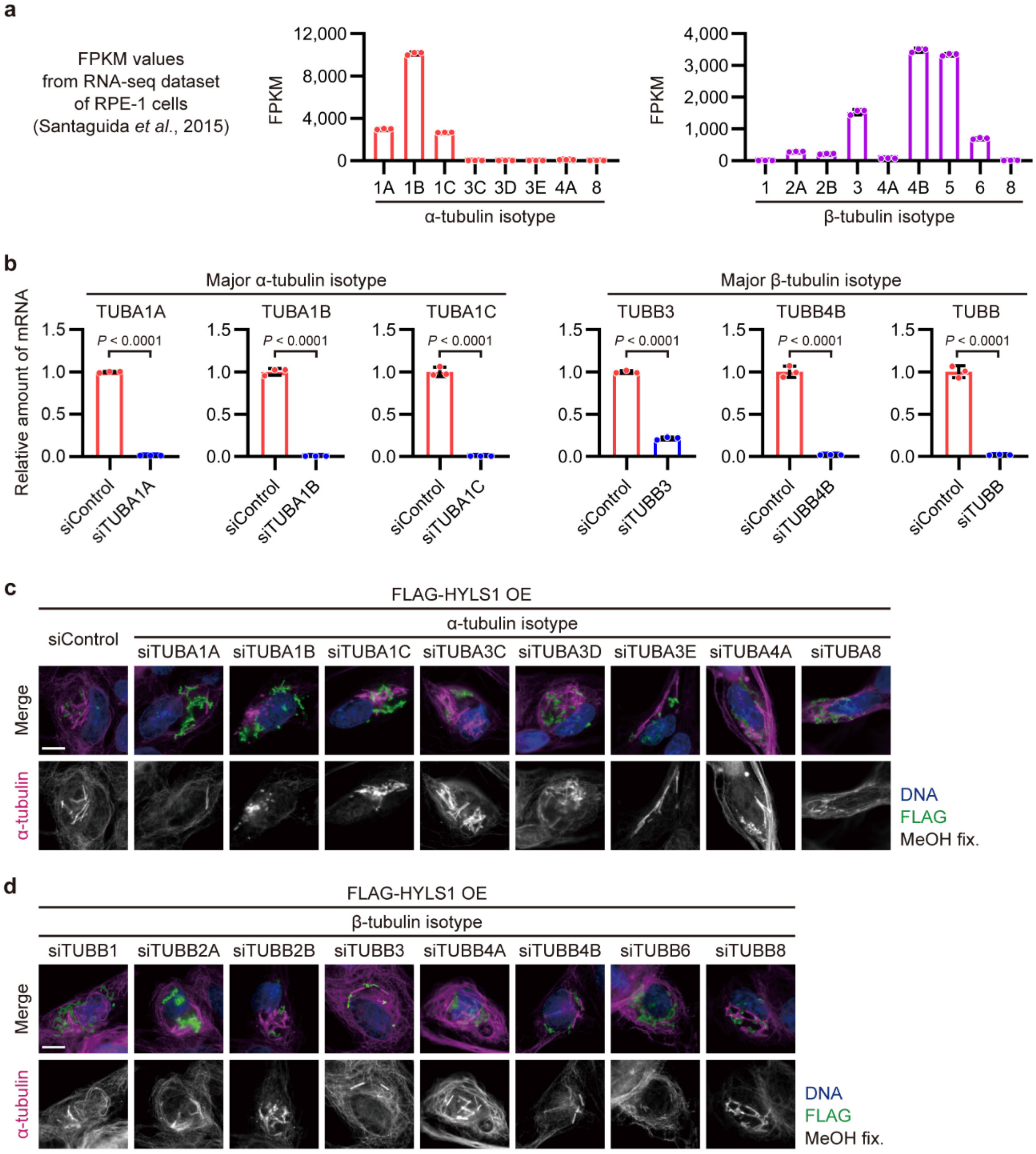

Supplementary Fig. 8 The β-tubulin isotype TUBB is specifically required for the HYLS1-dependent assembly of tubulin-based superstructures.

a, FPKM values for human tubulin isotypes from an RNA-seq dataset of RPE-1 cells in a previous study (GSE60570). *n* = 3 biological replicates. Data for untreated samples were used. **b**, Quantitative PCR analyses of major tubulin isotypes in RPE-1 cells treated with siControl or siRNAs targeting each isotype. *n* = 3 biological replicates. **c**, **d**, Supplementary IF images related

to Fig. 4e. Scale bars: 10 µm. Data are represented as mean ± s.d.. *P* values were calculated by two-tailed unpaired Student's *t*-test (**b**). Source data are provided as a Source Data file.

Supplementary Fig. 9

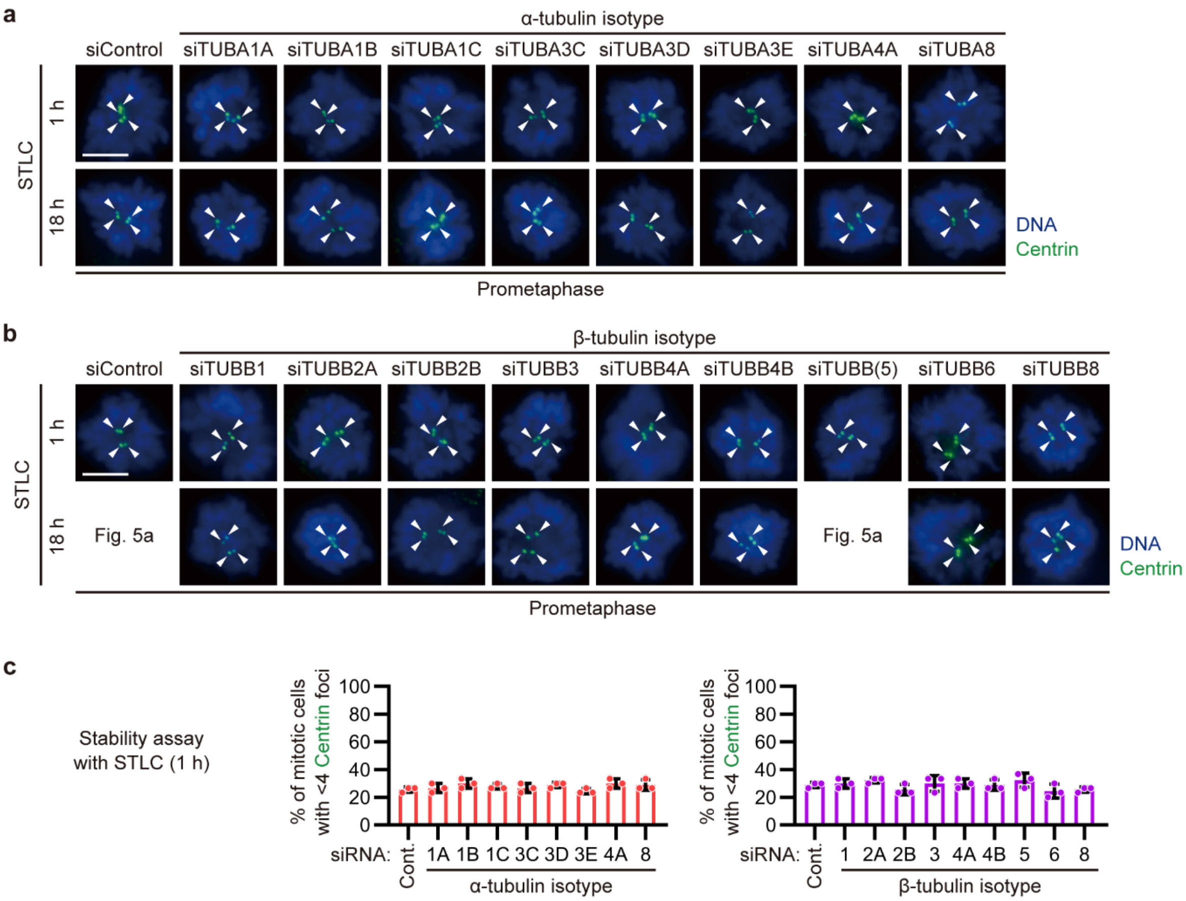

Supplementary Fig. 9 TUBB is specifically required for the assembly of stable centrioles.
a, **b**, Supplementary IF images related to Fig. 5a. Scale bars: 5 µm. Arrowheads: Centrin foci (centrioles). **c**, Supplementary quantification of frequency of mitotic cells with <4 Centrin foci related to Fig. 5b. Groups treated with STLC for 1 hour are shown. Cont.: control. n = 3 independent experiments, 30 cells each. Data are represented as mean ± s.d.. Source data are provided as a Source Data file.

Supplementary Fig. 10

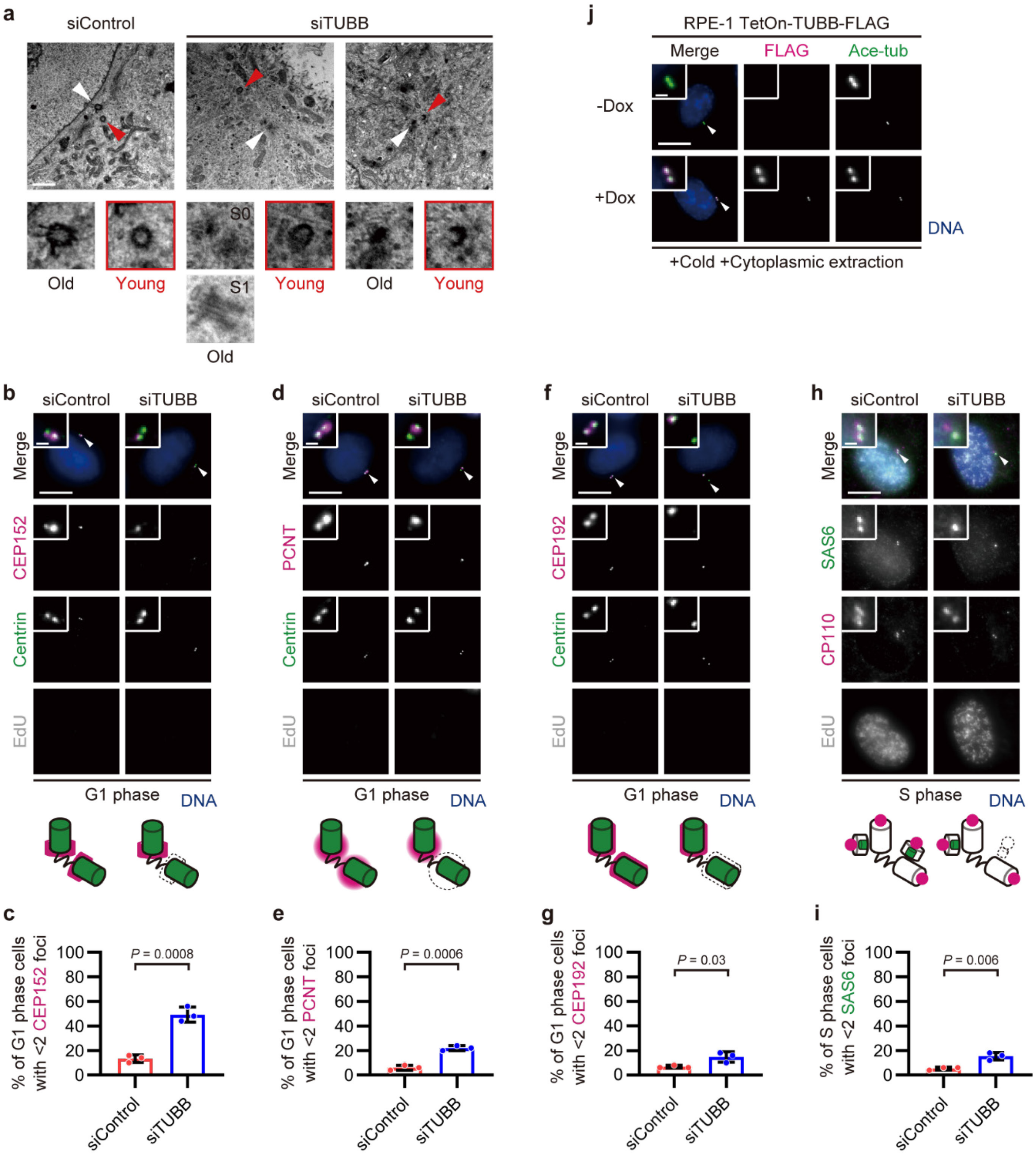

Supplementary Fig. 10 TUBB provides the structural integrity of centriole triplet microtubules.

a, Supplementary TEM images at low magnification related to Fig. 5c. The younger centrioles were distinguished by the absence of appendages. Scale bar: 1 μm. White arrowheads: older centrioles, red arrowheads: younger centrioles. S0: serial#0 (the same serial as the low

magnification image above), S1: serial#1 (adjacent serial). **b**, IF images of G1 phase RPE-1 cells transfected with siControl or siTUBB. Scale bars: 10 µm and 1 µm. Arrowheads: magnified areas. **c**, Quantification of frequency of G1 phase cells with <2 CEP152 foci in **b**. n = 3 independent experiments, 50 cells each. **d**, IF images of G1 phase RPE-1 cells transfected with siControl or siTUBB. Scale bars: 10 µm and 1 µm. Arrowheads: magnified areas. **e**, Quantification of frequency of G1 phase cells with <2 PCNT foci in **d**. n = 3 independent experiments, 50 cells each. **f**, IF images of G1 phase RPE-1 cells transfected with siControl or siTUBB. Scale bars: 10 µm and 1 µm. Arrowheads: magnified areas. **g**, Quantification of frequency of G1 phase cells with <2 CEP192 foci in **f**. n = 3 independent experiments, 50 cells each. **h**, IF images of S phase RPE-1 cells transfected with siControl or siTUBB. Scale bars: 10 µm and 1 µm. Arrowheads: magnified areas. **i**, Quantification of frequency of S phase cells with <2 SAS6 foci in **h**. n = 3 independent experiments, 50 cells each. **j**, IF images of RPE-1 TetOn-TUBB-FLAG cells in presence and absence of doxycycline. The cells were subjected to cold treatment and cytoplasmic extraction to depolymerize cytoplasmic microtubules. Scale bars: 10 µm and 1 µm. Arrowheads: magnified areas. Ace-tub: acetylated tubulin. Data are represented as mean ± s.d. P values were calculated by two-tailed unpaired Student's t-test (**c**, **e**, **g**, **i**). Source data are provided as a Source Data file.

Supplementary Fig. 11

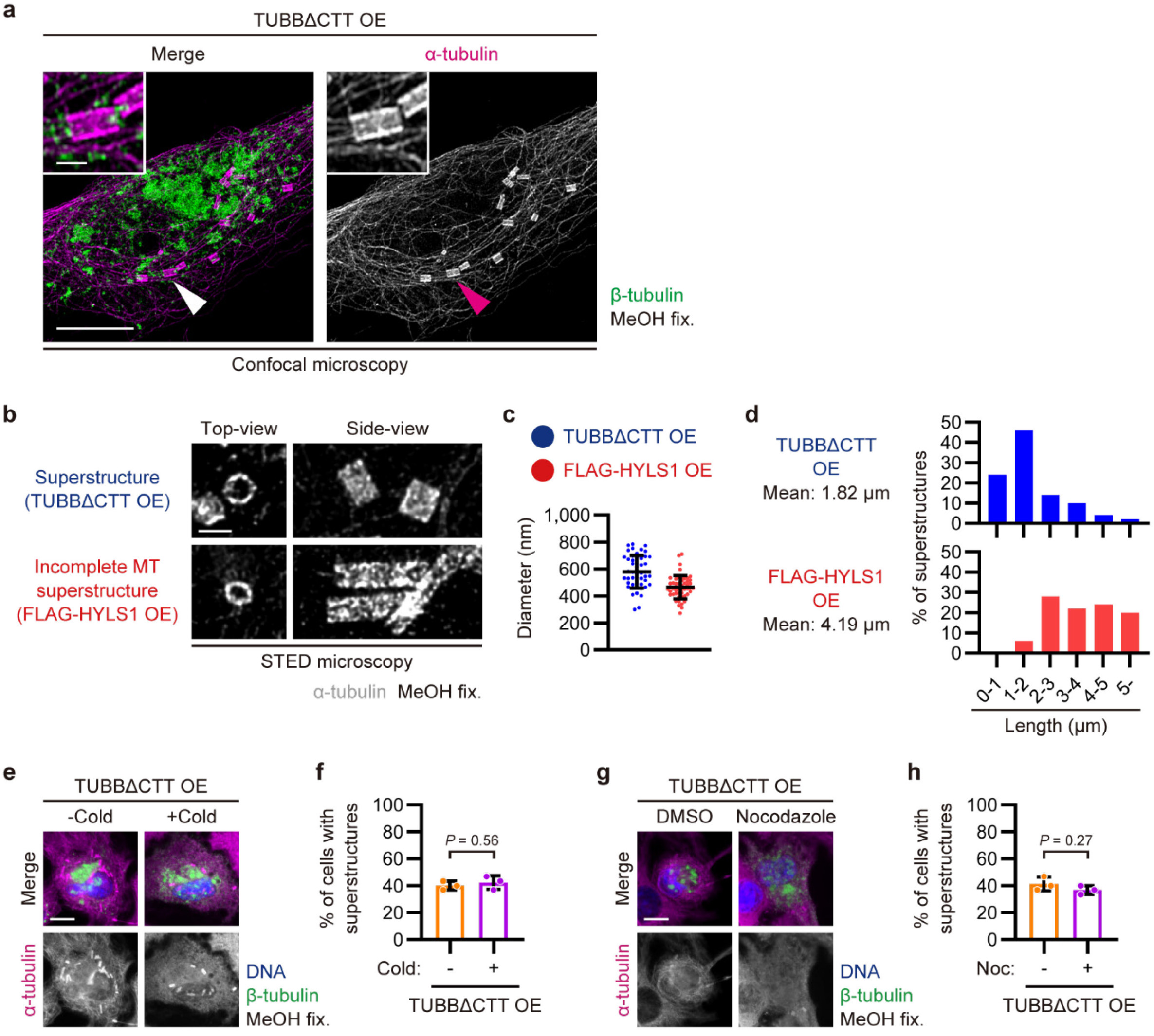

Supplementary Fig. 11 The unstructured C-terminal tail of TUBB prevents the assembly of incomplete microtubules.

a, Confocal microscopy images of RPE-1 cells transfected with pCMV-TUBBΔCTT. Scale bars: 10 µm and 1 µm. Arrowheads: magnified area. **b**, STED microscopy images of the tubulin-based superstructures in RPE-1 cells transfected with pCMV-TUBBΔCTT or pCMV-FLAG-HYLS1. Scale bar: 1 µm. **c**, Quantification of diameter of the tubulin-based superstructures in **b**. n = 50 superstructures. **d**, Quantification of length of the tubulin-based superstructures in **b**. n = 50 superstructures. **e**, IF images of RPE-1 cells transfected with pCMV-TUBBΔCTT and then incubated on ice for 1 hour before fixation. Scale bar: 10 µm. **f**, Quantification of frequency of interphase cells with the tubulin-based superstructures in **e**. n = 3 independent experiments, 30

cells each. **g**, IF images of RPE-1 cells transfected with pCMV-TUBBΔCTT and then treated with DMSO or nocodazole for 2 hours before fixation. Scale bar: 10 μm. **h**, Quantification of frequency of interphase cells with the tubulin-based superstructures in **g**. $n = 3$ independent experiments, 30 cells each. Data are represented as mean ± s.d.. *P* values were calculated by two-tailed unpaired Student's *t*-test (**f**, **h**). Source data are provided as a Source Data file.

Supplementary Fig. 12

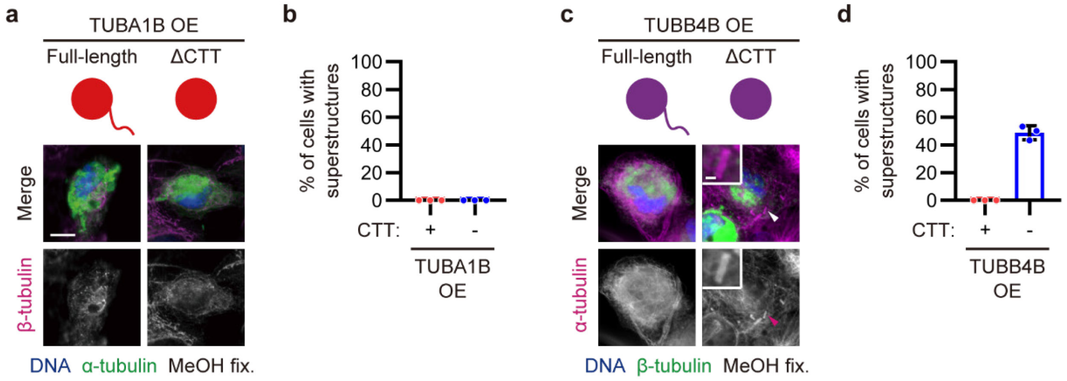

Supplementary Fig. 12 TUBB4BΔCTT, but not TUBA1BΔCTT, can also induce the assembly of incomplete microtubules.

a, IF images of RPE-1 cells transfected with pCMV-TUBA1B with or without the C-terminal tail (CTT). Scale bar: 10 µm. **b**, Quantification of frequency of interphase cells with tubulin-based superstructures similar to incomplete MT superstructures in **a**. $n = 3$ independent experiments, 30 cells each. **c**, IF images of RPE-1 cells transfected with pCMV-TUBB4B with or without the CTT. Scale bars: 10 µm and 1 µm. Arrowheads: magnified area. **d**, Quantification of frequency of interphase cells with tubulin-based superstructures similar to incomplete MT superstructures in **c**. $n = 3$ independent experiments, 30 cells each. Data are represented as mean ± s.d.. Source data are provided as a Source Data file.

Supplementary Fig. 13

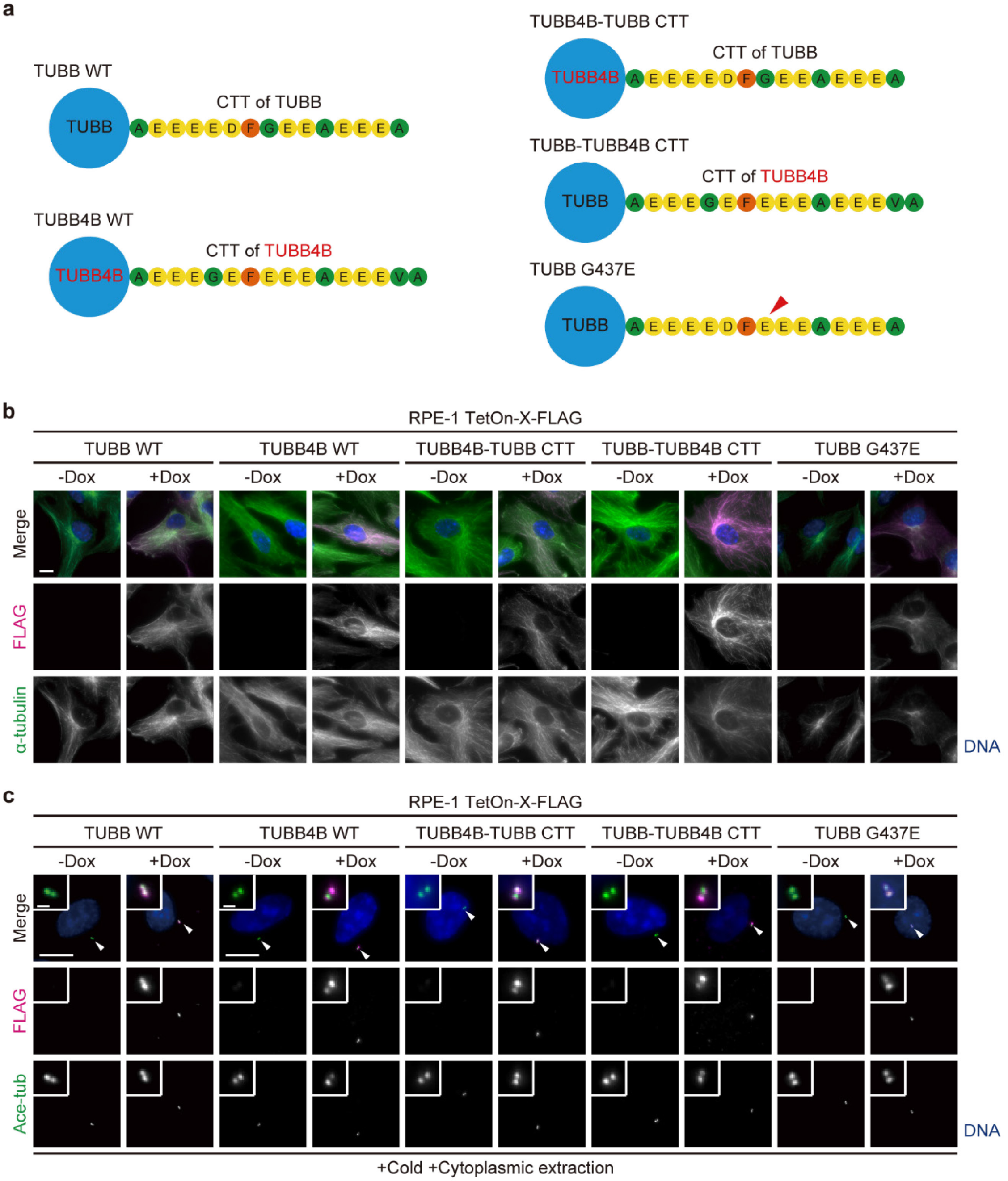

Supplementary Fig. 13 Generation of RPE-1 TetOn-X-FLAG cells.

a, Schematic of TUBB WT, TUBB4B WT, TUBB4B-TUBB CTT, TUBB-TUBB4B CTT, and TUBB G437E. CTT: C-terminal tail. **b**, IF images of RPE-1 TetOn-X-FLAG cells (X = TUBB WT, TUBB4B WT, TUBB4B-TUBB CTT, TUBB-TUBB4B CTT, or TUBB G437E) in presence and absence of doxycycline. Scale bar: 10 μm. **c**, IF images of RPE-1 TetOn-X-FLAG cells (X =

TUBB WT, TUBB4B WT, TUBB4B-TUBB CTT, TUBB-TUBB4B CTT, or TUBB G437E) in presence and absence of doxycycline. The cells were subjected to cold treatment and cytoplasmic extraction to depolymerize cytoplasmic microtubules. Scale bars: 10 µm and 1 µm. Arrowheads: magnified areas. Ace-tub: acetylated tubulin.

Supplementary Fig. 14

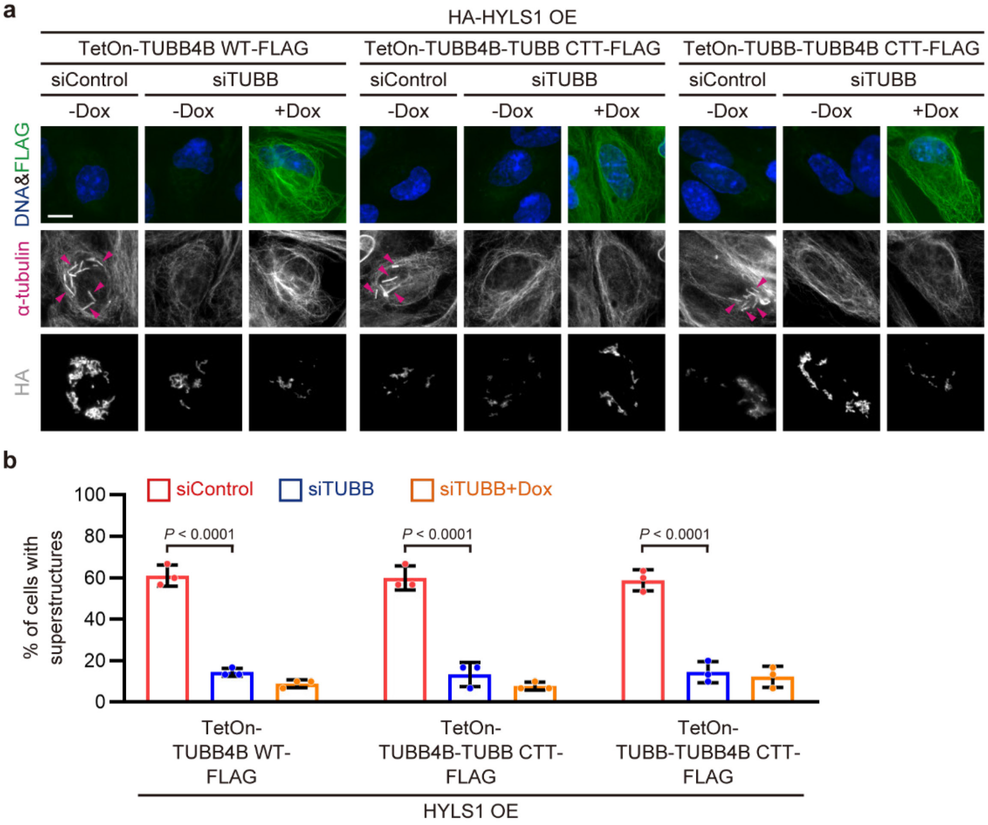

Supplementary Fig. 14 The TUBB-specific CTT, as well as the body of TUBB, is required for the HYLS1-dependent assembly of incomplete microtubules.

a, IF images of RPE-1 TetOn-X-FLAG cells (X = TUBB4B, TUBB4B-TUBB CTT, or TUBB-TUBB4B CTT) transfected with pCMV-HA-HYLS1 and siControl or siTUBB, and treated with doxycycline. Scale bar: 10 µm. Arrowheads: incomplete MT superstructures. **b**, Quantification of frequency of interphase cells with incomplete MT superstructures in **a**. n = 3 independent experiments, 30 cells each. Data are represented as mean ± s.d.. P values were calculated by one-way ANOVA with Tukey's multiple comparisons test (**b**). Source data are provided as a Source Data file.

Supplementary Fig. 15

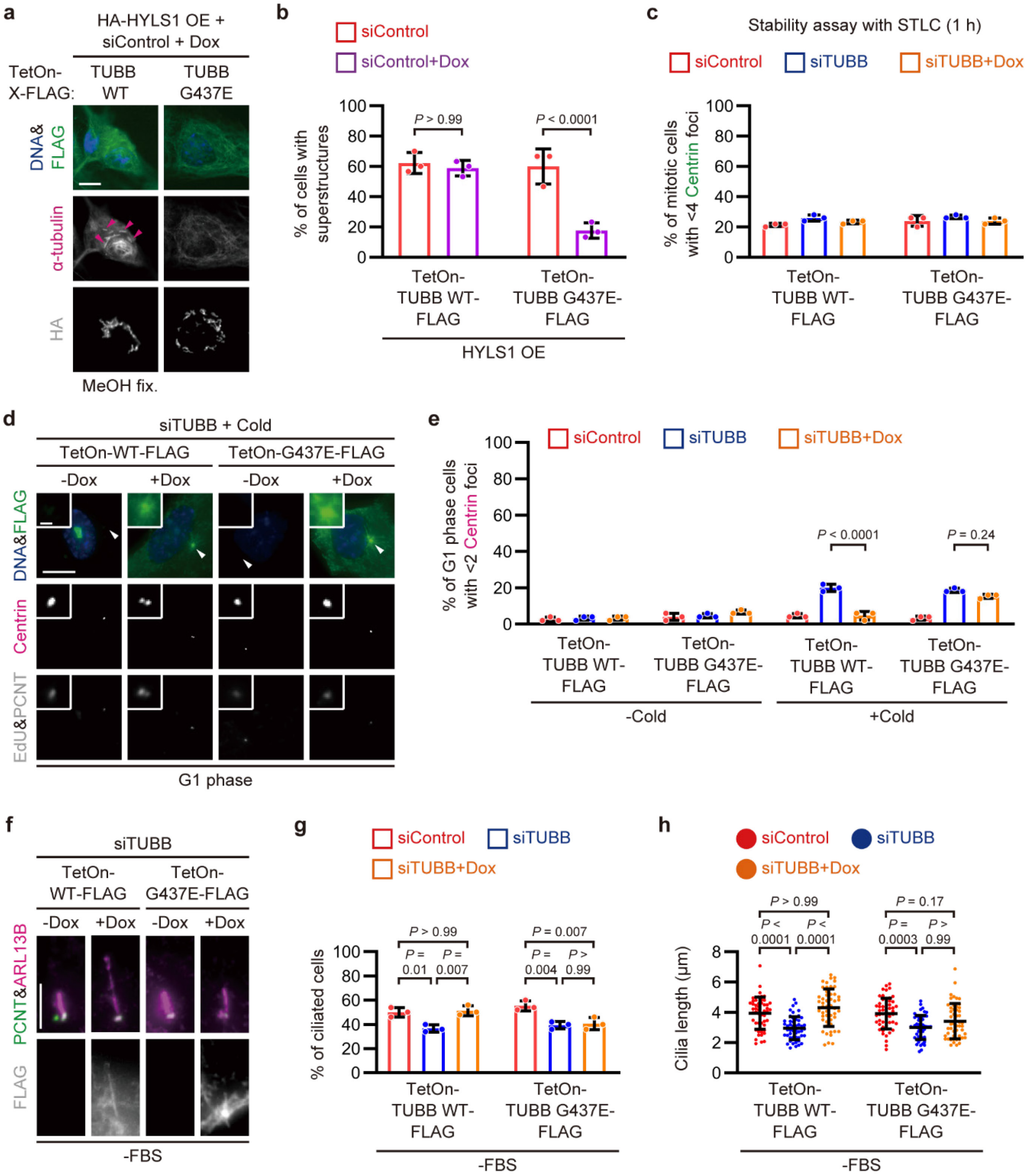

Supplementary Fig. 15 HYLS1 regulates the C-terminal tail of TUBB to promote the assembly of incomplete microtubules and stable centrioles.

a, Supplementary IF images related to Fig. 6f. Arrowheads: incomplete MT superstructures. Scale bar: 10 μm. **b**, Supplementary quantification of frequency of interphase cells with incomplete MT superstructures related to Fig. 6g. $n = 3$ independent experiments, 30 cells each. **c**, Supplementary

quantification of frequency of mitotic cells with <4 Centrin foci related to Fig. 6i. Groups treated with STLC for 1 hour are shown. *n* = 3 independent experiments, 50 cells each. **d**, Representative IF images of RPE-1 TetOn-X-FLAG cells (X = TUBB WT or G437E) subjected to centriole stability assay with cold treatment. Before incubation on ice, the cells were transfected with siControl or siTUBB and were treated with doxycycline. Scale bars: 10 µm and 1 µm. Arrowheads: magnified areas. **e**, Quantification of frequency of G1 phase cells with <2 Centrin foci in **d**. *n* = 3 independent experiments, 50 cells each. **f**, Representative IF images of serum-starved RPE-1 TetOn-X-FLAG cells (X = TUBB WT or G437E). The cells were transfected with siControl or siTUBB and were treated with doxycycline, followed by serum starvation for 48 hours. Scale bar: 5 µm. **g**, Quantification of frequency of interphase cells with primary cilia in **f**. *n* = 3 independent experiments, 50 cells each. **h**, Quantification of the length of primary cilia in **f**. *n* = 50 cells pooled from 3 independent experiments. Data are represented as mean ± s.d.. *P* values were calculated by one-way ANOVA with Tukey's multiple comparisons test (**b**, **e**, **g**) or Dunn's multiple comparisons test (**h**). Source data are provided as a Source Data file.

Supplementary Table 1 Plasmids generated in this study.

Plasmid	Vector	Insertion	Primer sequence for PCR (5'-3')	
pCMV5-FLAG-HYLS1	pCMV5-FLAG	HYLS1	F	TTCCTGCAGGTCGACATGGAAGAACTTCTACCTGA
			R	CCGGGATCAGGATCCTTAAGAAGGAGAAAGAGGGA
pCMV5-HA-HYLS1	pCMV5-HA	HYLS1	F	TTCCTGCAGGTCGACATGGAAGAACTTCTACCTGA
			R	CCGGGATCAGGATCCTTAAGAAGGAGAAAGAGGGA
pCMV5-FLAG-HYLS1 (*C. elegans*)	pCMV5-FLAG	HYLS1 (*C. elegans*)	F	TTCCTGCAGGTCGACATGGCCAATTTTACTACAG
			R	CCGGGATCAGGATCCTCAGTCAATATAAGGTCGTG
pCMV5-FLAG-HYLS1 (*D. melanogaster*)	pCMV5-FLAG	HYLS1 (*D. melanogaster*)	F	TTCCTGCAGGTCGACATGTCCCATCTGCCGCTCG
			R	CCGGGATCAGGATCCTCACTTCTTGAGTCCCATG
pCMV5-mScarlet-i-HYLS1	pCMV5-FLAG-HYLS1	mScarlet-i	F	GAATTGGCCGCCACCATGGTGAGCAAGGGCGAG
			R	TAGAAGTTCTTCCATTGCACCAGCTCCTGCACC
pCMV5-FLAG-HYLS1 (1-100 a.a.)	pCMV5-FLAG	HYLS1 (1-100 a.a.)	F	TTCCTGCAGGTCGACATGGAAGAACTTCTACCTGA
			R	CCGGGATCAGGATCCTTAGCGCAGCACCTTTCTCTT
pCMV5-FLAG-HYLS1 (1-150 a.a.)	pCMV5-FLAG	HYLS1 (1-150 a.a.)	F	TTCCTGCAGGTCGACATGGAAGAACTTCTACCTGA
			R	CCGGGATCAGGATCCTTAAACATCAAATGAAGATTC
pCMV5-FLAG-HYLS1 (1-200 a.a.)	pCMV5-FLAG	HYLS1 (1-200 a.a.)	F	TTCCTGCAGGTCGACATGGAAGAACTTCTACCTGA
			R	CCGGGATCAGGATCCTTACAGCTTTGGGAGAATAAAG
pCMV5-FLAG-HYLS1 (101-200 a.a.)	pCMV5-FLAG	HYLS1 (101-200 a.a.)	F	TTCCTGCAGGTCGACAGAAAGCCAGATGGGGAAG
			R	CCGGGATCAGGATCCTTACAGCTTTGGGAGAATAAAG
pCMV5-FLAG-HYLS1 (101-299 a.a.)	pCMV5-FLAG	HYLS1 (101-299 a.a.)	F	TTCCTGCAGGTCGACAGAAAGCCAGATGGGGAAG
			R	CCGGGATCAGGATCCTTAAGAAGGAGAAAGAGGGA
pCMV5-FLAG-HYLS1 (151-299 a.a.)	pCMV5-FLAG	HYLS1 (151-299 a.a.)	F	TTCCTGCAGGTCGACTCACAAAAATTTAACCTAC
			R	CCGGGATCAGGATCCTTAAGAAGGAGAAAGAGGGA
pCMV5-FLAG-HYLS1 (201-299 a.a.)	pCMV5-FLAG	HYLS1 (201-299 a.a.)	F	TTCCTGCAGGTCGACGACCAGTTAAGCCGAAACC
			R	CCGGGATCAGGATCCTTAAGAAGGAGAAAGAGGGA
pCMV5-FLAG-HYLS1 D211G	pCMV5-FLAG-HYLS1		F	AAGACAGGCCGGGTAGCCCGGTATTTTG
			R	TACCCGGCCTGTCTTGCCCCGGTTTCGG
pCMV5-FLAG-TUBB	pCMV5-FLAG	TUBB	F	TTCCTGCAGGTCGACATGAGGGAAATCGTGCAC
			R	CCGGGATCAGGATCCTTTAGGCCTCCTCTTCGGC
pCMV5-TUBB	pCMV5	TUBB	F	GAATTGGCCGCCACCATGAGGGAAATCGTGCAC
			R	CCGGGATCAGGATCCTTAGGCCTCCTCTTCGGCCTC
pCMV5-TUBB (1-429 a.a.)	pCMV5	TUBB (1-429 a.a.)	F	GAATTGGCCGCCACCATGAGGGAAATCGTGCAC
			R	CCGGGATCAGGATCCTTAGGTGGCATCCTGGTACTG
pCMV5-TUBA1B	pCMV5	TUBA1B	F	GAATTGGCCGCCACCATGCGTGAGTGCATCTCC
			R	CCGGGATCAGGATCCTTAGTATTCCTCTCCTTC
pCMV5-TUBA1B (1-439 a.a.)	pCMV5	TUBA1B (1-439 a.a.)	F	GAATTGGCCGCCACCATGCGTGAGTGCATCTCC
			R	CCGGGATCAGGATCCTTAATCCACACCAACCTCCTC
pCMV5-TUBB4B	pCMV5	TUBB4B	F	GAATTGGCCGCCACCATGAGGGAAATCGTGCAC
			R	CCGGGATCAGGATCCTTAGGCCACCTCCTCCTCAGC
pCMV5-TUBB4B (1-429 a.a.)	pCMV5	TUBB4B (1-429 a.a.)	F	GAATTGGCCGCCACCATGAGGGAAATCGTGCAC
			R	CCGGGATCAGGATCCTTATGTGGCATCCTGGTACTG
pRetroX-TRE3G-mNG-HYLS1	pRetroX-TRE3G-mNeonGreen	HYLS1	F	TGGTGCAGGCGGCCGCATGGAAGAACTTCTACCTG
			R	CTACCCGGTAGAATTCTTAAGAAGGAGAAAGAGGG
pRetroX-TRE3G-mNG-HYLS1 RR	pRetroX-TRE3G-mNG-HYLS1		F	GACCGACGAATCCATCATCAGTGAATCAGAATCTG
			R	GATGGATTCGTCGGTCACTAATACTTCCCCATCTG
pRetroX-TRE3G-mNG-HYLS1 D211G RR	pRetroX-TRE3G-mNG-HYLS1 RR		F	AAGACAGGCCGGGTAGCCCGGTATTTTG
			R	TACCCGGCCTGTCTTGCCCCGGTTTCGG
pRetroX-TRE3G-TUBB-FLAG	pRetroX-TRE3G-FLAG	TUBB	F	ATCCATCGATGCCACCATGAGGGAAATCGTGCACAT
			R	TTCGGGCCCTTACTTGTCATCGTCGTCCTTGTAGTCGG CCTCCTCTTCGGCCTCCT
pRetroX-TRE3G-TUBB RR-FLAG	pRetroX-TRE3G-TUBB-FLAG		F	GTAAAATTCGGGAGGAGTACCCTGATCGCATCATGAA TA
			R	TACTCCTCCCGAATTTTACTGATAAGGAGAGTGCCCAT T
pRetroX-TRE3G-TUBB G437E RR-FLAG	pRetroX-TRE3G-TUBB RR-FLAG		F	GATTTCGAGGAGGAGGCCGAAGAGGAG
			R	CTCCTCCTCGAAATCCTCCTCCTCTTC
pRetroX-TRE3G-TUBB4B-FLAG	pRetroX-TRE3G-FLAG	TUBB4B	F	CTTATACTTGGATCCATCGATGCCACCATGAGGGAAA TCGTGCACTT
			R	TACCCGGTAGAATTCGGGCCCTTACTTGTCATCGTCG TCCTTGTAGTCGGCCACCTCCTCCTCAGCCT

Plasmid	Vector	Insertion	Primer sequence for PCR (5'-3')	
pRetroX-TRE3G-TUBB4B-TUBB CTT-FLAG	pRetroX-TRE3G-TUBB CTT-FLAG	TUBB4B (1-429 a.a.)	F	TCCATCGATGCCACCATGAGGGAAATCGTGCAC
			R	CTCCTCCTCTTCTGCTGTGGCATCCTGGTACTG
pRetroX-TRE3G-TUBB-TUBB4B CTT-FLAG	pRetroX-TRE3G-TUBB4B CTT-FLAG	TUBB (1-429 a.a.)	F	TCCATCGATGCCACCATGAGGGAAATCGTGCAC
			R	GCCCTCCTCCTCGGCGGTGGCATCCTGGTACTG
pQCXIZ-TUBB-mNG	pQCXIZ-mNeonGreen	TUBB	F	CGCGGCCGCGCCACCATGAGGGAAATCGTGCAC
			R	ACCTGCACCAGCTCCGGCCTCCTCTTCGGCCTC
pBluescript-HYLS1 N ter.	pBluescript	HYLS1 N ter.	F	GGTATCGATAAGCTTAGACAGTAGAAAGCAGGAGT
			R	CGCTCTAGAACTAGTCAGAGACTGTTTCTGAAGGG
pBluescript-HygR-mCherry-HYLS1 N ter.	pBluescript-HYLS1 N ter.	HygR-mCherry	F	ATGAAGCCTGAACTCACCGC
			R	GGCGCCTGCACCGGATCCCT
pX330-hSpCas9-sgHYLS1	pX330-hSpCas9	sgHYLS1	F	CACCGCTGCCAACATTCGTTCTTC
			R	AAACGAAGAACGAATGTTGGCAGC

Supplementary Table 2 siRNAs used in this study.

**siRNA**	**Supplier**	**Cat#**	**Sense sequence** (5'-3')
siHYLS1#1	Thermo Fisher Scientific	s47731	GUAACAGAUGAGUCGAUUATT
siHYLS1#2	Thermo Fisher Scientific	s47732	GCCCGGUAUUUUGAGUACATT
siTUBA1A#1	Thermo Fisher Scientific	s15400	GGUUAAUAGGUCAAAUUGUTT
siTUBA1A#2	Thermo Fisher Scientific	s15401	GUUGUGGUCUGAUCAGUUATT
siTUBA1B#1	Thermo Fisher Scientific	s20288	CAUCAUAUCUCAAAGUAAATT
siTUBA1B#2	Thermo Fisher Scientific	s20289	AAAGCUUUCUGGUUAGAUUTT
siTUBA1C#1	Thermo Fisher Scientific	s195488	ACCUGUGUGCUGUACUUUUTT
siTUBA1C#2	Thermo Fisher Scientific	s224963	GUCUUGGAACUGUCUUAUUTT
siTUBA3C#1	Thermo Fisher Scientific	s194842	CGGUCAUCUCAGCCGAGAATT
siTUBA3C#2	Thermo Fisher Scientific	s194843	AGGAGAUCGUCGACCUGGUTT
siTUBA3D	Thermo Fisher Scientific	s195595	GCCUGGACCAUAAGUUCGATT
siTUBA3E#1	Thermo Fisher Scientific	s41356	CAAGCGCACUAUCCAGUUUTT
siTUBA3E#2	Thermo Fisher Scientific	s195587	GCACUAUCCAGUUUGUGGATT
siTUBA4A#1	Thermo Fisher Scientific	s14497	GCCGCAACCUAGACAUCGATT
siTUBA4A#2	Thermo Fisher Scientific	s14498	CUAUGCCCGUGGUCACUAUTT
siTUBA8#1	Thermo Fisher Scientific	s28671	GGAUGUGAAUGUCGCUAUUTT
siTUBA8#2	Thermo Fisher Scientific	s28672	GGGCCGUCAUGAUAGAUCUTT
siTUBB1#1	Thermo Fisher Scientific	s37479	CAGUCUUGGUGGACCUAGATT
siTUBB1#2	Thermo Fisher Scientific	s37480	CAUUCGAUCUAGCAAAUUATT
siTUBB2A#1	Thermo Fisher Scientific	s14500	GUAUUGUCUGUGAAGGUAUTT
siTUBB2A#2	Thermo Fisher Scientific	s14501	UCCUCUAAAAAUUACAGUATT
siTUBB2B#1	Thermo Fisher Scientific	s51294	CAACCUGAGUUUUAAAAUATT
siTUBB2B#2	Thermo Fisher Scientific	s223916	GGUAAAAGCUCAAAUGAAUTT
siTUBB3#1	Thermo Fisher Scientific	s20296	GCAAGGUGCGUGAGGAGUATT
siTUBB3#2	Thermo Fisher Scientific	s20297	AAUCUAUUGCUGUCAGAUATT
siTUBB4A#1	Thermo Fisher Scientific	s20299	ACUUCUCUUUGAUCUCUGATT
siTUBB4A#2	Thermo Fisher Scientific	s20300	GGAGGUUAUCAGUGACGAATT
siTUBB4B#1	Thermo Fisher Scientific	s20301	AGACCUACUGCAUUGAUAATT
siTUBB4B#2	Thermo Fisher Scientific	s20302	ACAACUUCGUUUUCGGUCATT
siTUBB#1	Thermo Fisher Scientific	s284	GCAAGAUCCGAGAAGAAUATT
siTUBB#2	Thermo Fisher Scientific	s283	GCACUCUCCUUAUCAGCAATT
siTUBB6#1	Thermo Fisher Scientific	s39180	GCAAGAUCCGUGAGGAGUUTT
siTUBB6#2	Thermo Fisher Scientific	s39182	GGAACAAAGACUAAAAACATT
siTUBB8#1	Thermo Fisher Scientific	s51282	ACAACUUCAUCUUCGGUCATT
siTUBB8#2	Thermo Fisher Scientific	s196882	AACUCUCCUUUUCUAGGUATT
siControl	Thermo Fisher Scientific	4390843	

Supplementary Table 3 Primers used in genomic PCR.

Figure	Target	Sequence (5'-3')	
Supplementary Fig. 1b	HYLS1	F	GACCTGAAGGTGGCAAGACA
		R	TTTCGGAGTCTTTGGGAGGC

Supplementary Table 4 Primers used in real-time PCR.

Figure	Target		Sequence (5'-3')
Supplementary Fig. 8b	TUBA1A	F	CTTCGTCTCCGCCATCAG
		R	TTGCCAATCTGGACACCA
	TUBA1B	F	CCCTAGCCACTATGCGTGA
		R	CGTGTTCCAGGCAGTAGAGC
	TUBA1C	F	CCCCTTCAAGTTCTAGTCATGC
		R	GCATTGCCAATCTGGACAC
	TUBB3	F	GCAACTACGTGGGCGACT
		R	CGAGGCACGTACTTGTGAGA
	TUBB4B	F	CTGCTGCTGTTTGTCTACTTCC
		R	CTGATCACCTCCCAAAACTTG
	TUBB	F	ATACCTTGAGGCGAGCAAAA
		R	TCACTGATCACCTCCCAGAAC
	GAPDH	F	TCCACTGGCGTCTTCACC
		R	GGCAGAGATGATGACCCTTTT
