# Supplementary material for: Molecular basis promoting centriole triplet microtubule assembly
Source: Nat Commun. 2024 Mar 22;15:2216. doi: 10.1038/s41467-024-46454-x (PMC10960023; doi:10.1038/s41467-024-46454-x)
Supplement: Supplementary file 3 — Description of Additional Supplementary Files [file 41467_2024_46454_MOESM3_ESM.pdf]

Description of Additional Supplementary Files

Supplementary Movie 1.

Original movie of the time-lapse images in Fig. 1h and Supplementary Fig. 2a. RPE-1 cells stably expressing TUBB-mNeonGreen transfected with pCMV-mScarlet-i-HYLS1. Green: mNeonGreen, magenta: mScarlet-i. 5-minute intervals.
