# Supplementary material for: Molecular basis promoting centriole triplet microtubule assembly
Source: Nat Commun. 2024 Mar 22;15:2216. doi: 10.1038/s41467-024-46454-x (PMC10960023; doi:10.1038/s41467-024-46454-x)
Supplement: Supplementary file 5 — Reporting Summary [file 41467_2024_46454_MOESM5_ESM.pdf]

nature portfolio

Corresponding author(s): Takumi Chinen, Shoji Hata, Daiju Kitagawa

Last updated by author(s): Feb 14, 2024

Reporting Summary

Nature Portfolio wishes to improve the reproducibility of the work that we publish. This form provides structure for consistency and transparency in reporting. For further information on Nature Portfolio policies, see our Editorial Policies and the Editorial Policy Checklist.

Statistics

For all statistical analyses, confirm that the following items are present in the figure legend, table legend, main text, or Methods section.

n/a	Confirmed	
☐	☒	The exact sample size (n) for each experimental group/condition, given as a discrete number and unit of measurement
☐	☒	A statement on whether measurements were taken from distinct samples or whether the same sample was measured repeatedly
☐	☒	The statistical test(s) used AND whether they are one- or two-sided<br>*Only common tests should be described solely by name; describe more complex techniques in the Methods section.*
☒	☐	A description of all covariates tested
☐	☒	A description of any assumptions or corrections, such as tests of normality and adjustment for multiple comparisons
☐	☒	A full description of the statistical parameters including central tendency (e.g. means) or other basic estimates (e.g. regression coefficient) AND variation (e.g. standard deviation) or associated estimates of uncertainty (e.g. confidence intervals)
☐	☒	For null hypothesis testing, the test statistic (e.g. F, t, r) with confidence intervals, effect sizes, degrees of freedom and P value noted<br>*Give P values as exact values whenever suitable.*
☒	☐	For Bayesian analysis, information on the choice of priors and Markov chain Monte Carlo settings
☒	☐	For hierarchical and complex designs, identification of the appropriate level for tests and full reporting of outcomes
☒	☐	Estimates of effect sizes (e.g. Cohen's d, Pearson's r), indicating how they were calculated

Our web collection on statistics for biologists contains articles on many of the points above.

Software and code

Policy information about availability of computer code

Data collection	Images were acquired by:<br>Cell Voyager CV1000 (Yokogawa Electric Corp)<br>Axioplan2 fluorescence microscope (Carl Zeiss)<br>TCS SP8 STED 3X system (Leica)<br>980 laser-scanning microscope with AiryScan2 (Carl Zeiss)<br>1200EX (JEOL)<br>BZ-X800 systems (Keyence)<br>Chemi Doc XRS+ (BioRad)<br>Real time qPCR was performed on:<br>LightCycler 480 (Roche)
Data analysis	Images were analyzed by using ImageJ (version 1.8.0).<br>Structural models were generated by ColabFold (version 1.4.0)<br>Structural models were modified and exported by UCSF Chimera (version 1.16).<br>Statistical analyses were performed using GraphPad Prism (version 9.3.1) or Microsoft Office Excel (version 2401).<br>We have not developed any custom codes in this study.

For manuscripts utilizing custom algorithms or software that are central to the research but not yet described in published literature, software must be made available to editors and reviewers. We strongly encourage code deposition in a community repository (e.g. GitHub). See the Nature Portfolio guidelines for submitting code & software for further information.

Data

Policy information about availability of data

All manuscripts must include a data availability statement. This statement should provide the following information, where applicable:

- Accession codes, unique identifiers, or web links for publicly available datasets
- A description of any restrictions on data availability
- For clinical datasets or third party data, please ensure that the statement adheres to our policy

Source data are provided with this paper. All data used to generate graphs and all pictures showing uncropped gels or blots accompany this manuscript in the source data file. Underlying image data are available from the corresponding authors on reasonable request.
The Chronos scores analyzed in this study (DepMap Public 21Q3 CRISPR_gene_effect) are available on the Cancer Dependency Map (DepMap) website (https://depmap.org/portal/download/all/?releasename=DepMap+Public+21Q3&filename=CRISPR_gene_effect.csv).
The RNA-seq data reanalyzed in this study are available under the accession codes GSE60570 (https://www.ncbi.nlm.nih.gov/geo/query/acc.cgi?acc=GSE60570).

Research involving human participants, their data, or biological material

Policy information about studies with human participants or human data. See also policy information about sex, gender (identity/presentation), and sexual orientation and race, ethnicity and racism.

Reporting on sex and gender	N/A
Reporting on race, ethnicity, or other socially relevant groupings	N/A
Population characteristics	N/A
Recruitment	N/A
Ethics oversight	N/A

Note that full information on the approval of the study protocol must also be provided in the manuscript.

Field-specific reporting

Please select the one below that is the best fit for your research. If you are not sure, read the appropriate sections before making your selection.

☒ Life sciences ☐ Behavioural & social sciences ☐ Ecological, evolutionary & environmental sciences

For a reference copy of the document with all sections, see nature.com/documents/nr-reporting-summary-flat.pdf

Life sciences study design

All studies must disclose on these points even when the disclosure is negative.

Sample size	The number of experiments and sample size were chosen in order to make sure they will be sufficient for statistical analysis. In principle, three independent experiments were conducted and at least n= 30 were taken for each time, to the extent that it was realistically possible to quantify the results. For TEM analyses, where it was not feasible to obtain more than 30 objects, all that could be observed were subjected to the quantification. No statistical method was used to predetermine the correct sample size.
Data exclusions	No data were excluded for all analyses.
Replication	All experiments were successfully replicated at least three times.
Randomization	This study used cultured cell lines which are homogenous and isogenic, therefore no randomization was used.
Blinding	The investigators were not blinded to allocation during experiments and outcome assessment since blinding is technically and practically not feasible in this type of cell biological research. However, to reduce potential human bias, the quantifications were done by multiple authors and the results were reconciled.

Reporting for specific materials, systems and methods

We require information from authors about some types of materials, experimental systems and methods used in many studies. Here, indicate whether each material, system or method listed is relevant to your study. If you are not sure if a list item applies to your research, read the appropriate section before selecting a response.

Materials & experimental systems

n/a	Involved in the study	
	☒	Antibodies
	☒	Eukaryotic cell lines
☒		Palaeontology and archaeology
☒		Animals and other organisms
☒		Clinical data
☒		Dual use research of concern
☒		Plants

Methods

n/a	Involved in the study	
☒		ChIP-seq
☒		Flow cytometry
☒		MRI-based neuroimaging

Antibodies

Antibodies used

The following primary antibodies were used in this study:
Rabbit polyclonal antibodies against:
RFP/mCherry [MBL, PM005, Lot 046, IF 1:500, IB 1:1000]
CEP97 [Novus Biologicals, NBP1-83591, Lot A107064, IF 1:500]
α-tubulin [MBL, PM054, Lot 007, IF 1:500, IB 1:1000]
β-tubulin [Thermo Fisher Scientific, PA5-16863, Lot XG3654589, IF 1:500]
acetylated tubulin [Abcam, ab179484, IF 1:500]
FLAG [Sigma, F7425, Lot 086M4803V, IF 1:500]
HA [Abcam, ab9110, IF 1:1000]
PCNT [Abcam, ab4448, IF 1:1000]
HYLS1 [Novus Biologicals, NBP1-56899, Lot QC25211-90821, IB 1:1000]
CEP152 [Bethyl Laboratories, A302-480A, IF 1:1000]
CEP192 [Bethyl Laboratories, A302-324A, IF 1:1000]
CP110 [Proteintech, 12780-1-AP, IF 1:500]
ANKRD26 [GeneTex, GTX128255, Lot 41458, IF 1:1000]
CEP44 [Proteintech, 24457-1-AP, IF 1:500]
CEP57 [GeneTex, GTX115931, Lot 40289, IF 1:500]
CEP63 [Proteintech, 16268-1-AP, IF 1:500]
CEP135 [Abcam, ab196809, Lot GR199116-6, IF 1:500]
CEP295 [Sigma, HPA038596, Lot R33456, IF 1:500]
CPAP [Proteintech, 11517-1-AP, IF 1:500]
PLK1 [Bethyl Laboratories, A300-251A, IF 1:500]
ARL13B [Proteintech, 17711-1-AP, IF 1:500]
Goat polyclonal antibody against:
PCNT [Santa Cruz Biotechnology, sc-28145, Lot C1716, IF 1:500]
Mouse monoclonal antibodies against:
Centrin [Merck Millipore, 04-1624, Clone 20H5, IF 1:1000]
γ-tubulin [Sigma, T5326, Clone GTU88, IF 1:1000]
α-tubulin [Sigma, T5168, Clone B-5-1-2, IF 1:1000]
acetylated tubulin [Sigma, T7451, Clone 6-11B-1, IF 1:500]
polyglutamylation modification [AdipoGen, AG-20B-0020-C100, Clone GT335, IF 1:500]
monoglycylated tubulin [Merck Millipore, MABS277, Clone TAP952, IF 1:500]
polyglycylated tubulin [Merck Millipore, MABS276, Clone AXO49, IF 1:500]
FLAG [Sigma, F1804, Clone M2, IF 1:1000, IB 1:1000]
HA [Biolegend, 901501, Clone 16B12, IF 1:500]
SAS6 [Santa Cruz Biotechnology, sc-81431, IF 1:500]
HSP90 [BD Biosciences, 610419, IB 1:2000]
EB1 [BD Biosciences, 610534, Clone 5/EB1, IF 1:500]
Rat monoclonal antibodies against:
HA [Merck, 11867423001, Clone 3F10, IF 1:500]
Centrin 2 [BioLegend, 698602, Clone W16110A, IF 1:500]

The following secondary antibodies were used in this study:
Alexa Fluor 488 donkey anti-mouse IgG (H+L) (Thermo Fisher Scientific, A32766, IF 1:500)
Alexa Fluor 488 donkey anti-rabbit IgG (H+L) (Thermo Fisher Scientific, A32790, IF 1:500)
Alexa Fluor 488 donkey anti-rat IgG (H+L) (Thermo Fisher Scientific, A48269, IF 1:500)
Alexa Fluor 555 donkey anti-mouse IgG (H+L) (Thermo Fisher Scientific, A32773, IF 1:500)
Alexa Fluor 555 donkey anti-rabbit IgG (H+L) (Thermo Fisher Scientific, A32794, IF 1:500)
Alexa Fluor 647 donkey anti-mouse IgG (H+L) (Thermo Fisher Scientific, A32787, IF 1:500)
Alexa Fluor 647 donkey anti-rabbit IgG (H+L) (Thermo Fisher Scientific, A32795, IF 1:500)
Alexa Fluor 647 donkey anti-goat IgG (H+L) (Thermo Fisher Scientific, A32849, IF 1:500)
Alexa Fluor 647 donkey anti-rat IgG (H+L) (Thermo Fisher Scientific, 48272, IF 1:500)

HRP-conjugated goat polyclonal antibodies against mouse IgG (Promega, W402B, IB 1:5000)
HRP-conjugated goat polyclonal antibodies against rabbit IgG (Promega, W401B, IB 1:5000).

Validation

Each primary antibody was sufficiently validated in human cells. Details are below.

The following antibodies were validated by knockdown experiments and used in this study:
Rabbit polyclonal antibodies against;
RFP/mCherry (MBL): applications: WB, IF, IHC.
HYLS1 (Novus Biologicals): application: WB.

The following antibodies were previously validated, used, and published by our lab (Ohta et al., 2014; Shiratsuchi et al., 2015; Tsuchiya et al., 2016; Ohta et al., 2018; Watanabe et al., 2019; Yamamoto & Kitagawa, 2019; Yoshiba et al., 2019; Takao et al., 2019; Chinen et al., 2020; Takeda et al., 2020; Chinen et al., 2021; Ito et al., 2021; Yamamoto et al., 2021) and other labs:
Rabbit polyclonal antibodies against:
α-tubulin (MBL): applications: WB, IP, IF. Used in 30 publications according to the supplier.
HA (Abcam): applications: ChIP, IP, ELISA, WB, IF, FC. Used in 1070 publications according to the supplier.
PCNT (Abcam): application: IF. Used in 487 publications according to the supplier.
CEP152 (Bethyl Laboratories): application: IP. Used in 29 publications according to the supplier.
CEP192 (Bethyl Laboratories): applications: IP, WB. Used in 31 publications according to the supplier.
CP110 (Proteintech): applications: WB, IP, IHC, IF, FC, ELISA. Used in 133 publications according to the supplier.
CEP57 (GeneTex): applications: WB, IF, IHC, IP. Used in 7 publications according to the supplier.
CEP63 (Proteintech): applications: WB, IP, IF, ELISA. Used in 13 publications according to the supplier.
CEP135 (Abcam): application: WB. Used in 2 publications according to the supplier.
CEP295 (Sigma): applications: IF, IHC. Used in 3 publications according to the supplier.
CPAP (Proteintech): applications: WB, IHC, IF, ELISA. Used in 46 publications according to the supplier.
PLK1 (Bethyl Laboratories): applications: IF, IP, WB. Used in 13 publications according to the supplier.
Mouse monoclonal antibodies against:
Centrin (Merck Millipore): applications: IF, IP, WB. Used in 172 publications according to the supplier.
γ-tubulin (Sigma): applications: ARR, ELISA, IF, IP, WB. Used in 335 publications according to the supplier.
α-tubulin (Sigma): applications: IF, RIA, WB. Used in 3640 publications according to the supplier.
acetylated tubulin (Sigma): applications: DB, EM, IHC, RIA, WB. Used in 1077 publications according to the supplier.
polyglutamylation modification (AdipoGen): applications: EM, IF, IHC, IP, WB. Used in 20 publications according to the supplier.
FLAG (Sigma): applications: IB, IP, IHC, IF. Used in 8801 publications according to the supplier.
HA (Biolegend): applications: WB, FC, IF, IP. Used in 577 publications according to the supplier.
SAS6 (Santa Cruz Biotechnology): applications: WB, IP, IF. Used in 78 publications according to the supplier.
HSP90 (BD Biosciences): applications: WB, IF, IP, IHC. Used in more than 5 publications according to the supplier.
EB1 (BD Biosciences): applications: WB, IF, IHC, IP. Used in more than 5 publications according to the supplier.

The following antibodies were previously used and published by other labs:
Rabbit polyclonal antibodies against;
CEP97 (Novus Biologicals): applications: WB, IF, IHC. Used in 2 publications according to the supplier.
β-tubulin (Thermo Fisher Scientific): applications: WB, IHC, IF. Used in 18 publications according to the supplier.
acetylated tubulin (Abcam): applications: WB, IF, IP, IHC, FC. Used in 34 publications according to the supplier.
FLAG (Sigma): applications: DB, IF, IP, WB. Used in 2792 publications according to the supplier.
ANKRD26 (GeneTex): applications: WB, IF. Used in 8 publications according to the supplier.
CEP44 (Proteintech): applications: WB, IP, IHC, IF, ELISA. Used in 5 publications according to the supplier.
ARL13B (Proteintech): applications: WB, IP, IHC, IF, FC, ELISA. Used in 686 publications according to the supplier.
Goat polyclonal antibody against;
PCNT (Santa Cruz Biotechnology): applications: WB, IF, IHC, ELISA. Used in 4 publications according to the supplier.
Mouse monoclonal antibodies against;
monoglycylated tubulin (Merck Millipore): applications: DB, IF, WB. Used in 9 publications according to the supplier.
polyglycylated tubulin (Merck Millipore): applications: DB, IF, WB. Used in 10 publications according to the supplier.
Rat monoclonal antibodies against;
HA (Merck): applications: DB, ELISA, ICC, IP, WB. Used in 33 publications according to the supplier.
Centrin 2 (Biolegend): applications: WB, IF. Used in 1 publication according to the supplier.

Eukaryotic cell lines

Policy information about cell lines and Sex and Gender in Research

Cell line source(s)

HeLa and HEK293 cells were obtained from the European Collection of Authenticated Cell Cultures (ECACC).
RPE-1 cells were obtained from the American Type Culture Collection (ATCC).
HEK GP2-293 cells were obtained from Clontech.

Authentication

HeLa, HEK293, RPE-1, or HEK GP2-293 cells were authenticated by the suppliers via short tandem repeat profiling.

Mycoplasma contamination

It was confirmed that the cells were not contaminated with mycoplasma using TaKaRa PCR Mycoplasma Detection Set (TaKaRa, 6601).

Commonly misidentified lines<br>(See ICLAC register)	No cell lines in the ICLAC database were used in this study.
